# Treatment duration as a surrogate endpoint to evaluate the efficacy of crizotinib in sequential therapy for patients with advanced ALK‐positive non‐small cell lung cancer: A retrospective, real‐world study

**DOI:** 10.1002/cam4.2420

**Published:** 2019-08-13

**Authors:** Guangjian Yang, Di Ma, Haiyan Xu, Lu Yang, Junling Li, Puyuan Xing, Xuezhi Hao, Yan Wang

**Affiliations:** ^1^ Department of Medical Oncology National Cancer Center/National Clinical Research Center for Cancer/Cancer Hospital Chinese Academy of Medical Sciences and Peking Union Medical College Beijing China; ^2^ Department of Comprehensive Oncology National Cancer Center/National Clinical Research Center for Cancer/Cancer Hospital Chinese Academy of Medical Sciences and Peking Union Medical College Beijing China

**Keywords:** anaplastic lymphoma kinase inhibitor, crizotinib, crizotinib continuation beyond progressive disease, non‐small cell lung cancer, treatment duration

## Abstract

**Objectives:**

Crizotinib has demonstrated good efficacy in patients with anaplastic lymphoma kinase (*ALK*)‐positive non‐small cell lung cancer (NSCLC). Continuing crizotinib therapy beyond progressive disease (CBPD) can achieve ongoing survival benefit in real‐world clinical practice. In terms of survival, progression‐free survival (PFS), the most commonly used endpoint in efficacy evaluations, may not provide accurate information on the impact of this intervention when crizotinib is administered in sequential therapy.

**Materials and Methods:**

A single‐center, retrospective study of 201 *ALK*‐positive advanced NSCLC patients was conducted to analyze the PFS, overall survival (OS), and treatment duration (TD) of crizotinib. The correlations between the TD of crizotinib and OS in CBPD and non‐CBPD groups of patients were compared.

**Results:**

All patients were treated with crizotinib, 150 of whom eventually developed progressive disease (PD). The median PFS1 and PFS2 were 13.2 months and 10.5 months, respectively. The OS of the whole population was 50.5 months. The median TD was 20.7 months, which is shorter than direct PFS1 + PFS2. The TD of crizotinib in CBPD group was significantly longer than that in non‐CBPD group (median 39.7 vs 15.0 months,* P* < .001). TD correlated better with OS (*R* = .79) than PFS (*R* = .64) in the CBPD group.

**Conclusions:**

Crizotinib showed good efficacy in patients with *ALK*‐positive advanced NSCLC. Instead of PFS, treatment duration might be a more reasonable surrogate clinical endpoint in patients who received crizotinib in sequential therapy.

## Graphical abstract

Crizotinib showed good efficacy on ORR, PFS, and OS in Chinese ALK‐positive NSCLC patients. PFS is not accurate to be used to evaluate the true clinical efficacy of crizotinib. CBPD is a consideration when assessing sustained PFS benefit by crizotinib. TD in CBPD group was significantly longer than that in non‐CBPD group. TD instead of PFS correlates better with OS.

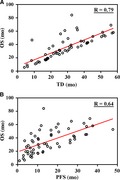

## 1 INTRODUCTION

Lung cancer is the leading cause of cancer deaths in the world. Non‐small cell lung cancer (NSCLC), which accounts for 80%‐85% of lung cancers, is the most common histological type.^1^ Anaplastic lymphoma kinase gene (*ALK*) rearrangement occurs in approximately 2%‐7% of NSCLC patients.^2^ As the first *ALK*‐targeted small‐molecule tyrosine kinase inhibitor (TKI), clinical data from the PROFILE 1014 and PROFILE 1007 phase III clinical trials comparing crizotinib with chemotherapy in patients with advanced NSCLC have demonstrated the progression‐free survival (PFS) benefit and the objective response rate (ORR) superiority of crizotinib in both first‐ and second‐line settings.^3^, ^4^ Consequently, crizotinib has become a standard of care in such patients.

Despite its promising efficacy, crizotinib‐pretreated patients ultimately develop progressive disease (PD) by acquiring resistance after less than 1 year as a result of secondary *ALK* mutations or activation of alternative oncogenic pathways.^5^, ^6^ Continuing crizotinib therapy beyond progressive disease (CBPD) may also impact survival outcomes and may provide an ongoing survival benefit for patients with advanced *ALK*‐positive NSCLC.^7^, ^8^ However, how CBPD will contribute to the efficacy of crizotinib in the real‐world clinical practice was unclear.

Patients who had already developed the initial Response Evaluation Criteria in Solid Tumors (RECIST)‐defined PD after primary crizotinib treatment (PFS1) will have nearly another 3‐6 months PFS (PFS2) when they continued receiving crizotinib therapy beyond PD as literature reported.^9^, ^10^ Since only a part of patients will have CBPD treatment, PFS1 + PFS2 will overestimate the efficacy of crizotinib. While using PFS, the most frequently used endpoint of efficacy in clinical trials will underestimate its efficacy. The same phenomenon will also be observed in epidermal growth factor receptor (EGFR)—TKI treatment. In terms of that, we think treatment duration (TD), which is defined as the time between crizotinib initiation and its discontinuation, may be a more reasonable efficacy endpoint of crizotinib, as well as other TKIs, in multilines of therapy.

To evaluate TD of crizotinib, and investigate the relationship between TD and PFS1, PFS2, OS in a large cohort of patients with advanced *ALK*‐positive NSCLC who received crizotinib, we conducted a single‐center, retrospective, real‐world cohort study and reported the exploratory outcomes.

## 2 MATERIALS AND METHODS

### 2.1 Patients

Two hundred and one patients with advanced *ALK*‐positive NSCLC (stage IIIB/IV) who were treated between August 2007 and November 2017 at the National Cancer Center/National Clinical Research Center for Cancer/Cancer Hospital, Chinese Academy of Medical Sciences and Peking Union Medical College, were enrolled in this study and their data were retrospectively analyzed. The patients’ clinical characteristics were obtained from their medical records, including the date when the metastatic disease was diagnosed, the date of crizotinib initiation, and the date of the initial RECIST‐defined PD and crizotinib discontinuation. Clinical outcomes were also collected, including the date of death and information on disease progression based on increases in lesion size, new lesion appearances, or other recorded progression.

Histological classification of lung cancer was based on the World Health Organization pathological criteria. Tumor samples were obtained by either surgical or diagnostic procedures. *ALK* mutation detection was carried out either by fluorescence in situ hybridization, polymerase chain reaction (PCR) or immunohistochemistry (IHC) analysis. IHC analysis was conducted using a monoclonal D5F3 antibody (Ventana Medical Systems, Tucson, AZ, USA) directed against *ALK*.

### 2.2 Treatment

All patients were treated with crizotinib 250 mg twice daily (adjusting the dose as needed) either as the first‐line or later‐line therapy. Clinical staging was determined according to the 7th edition of Union for International Cancer Control Tumor Node Metastasis classification.^11^ The tumor response was classified according to the RECIST version 1.1,^12^ as either complete response (CR), partial response (PR), stable disease (SD), or progressive disease (PD).

PFS1 with crizotinib therapy was defined as the time from crizotinib initiation to the first RECIST‐defined PD. TD of crizotinib was defined as the time between the initiation of crizotinib treatment and its subsequent discontinuation. Patients could continlue crizotinib treatment with administering additional local therapies including whole brain radiotherapy (WBRT), stereotactic radiotherapy (SRT), surgical resection, and local ablation beyond the initial RECIST‐defined PD by primary crizotinib targeted therapy depending on the specialists’ assessment of symptoms and imaging examinations. Three weeks of crizotinib treatment after initial PD was defined as the cutoff for dividing patients into a CBPD group or a non‐CBPD group (>3 vs ≤3 weeks, respectively).

The cutoff follow‐up time for the study was 22 April 2018. OS was measured from the date of diagnosis of metastatic disease to death or the last follow‐up. Patients without a known date of death were censored at the time of the last follow‐up.

### 2.3 Statistical analysis

The Kaplan‐Meier method was used to estimate the curves for PFS, TD, and OS. Pearson's Chi‐squared test was used to compare the significance of baseline differences between two groups of data. Significant differences between the different groups were determined by the log‐rank test. A *P* value < .05 was considered statistically significant. Statistical analyses were performed using SPSS^®^ software, version 20.0 (IBM Corp., Armonk, NY, USA).

## 3 RESULTS

### 3.1 Patient characteristics

Among the 201 patients enrolled in the study, 150 (74.6%) acquired resistance to crizotinib and developed the RECIST‐defined PD. The median age at diagnosis was 50 years (range, 24‐83 years). Ninety‐five patients (47.2%) were male and the majority (69.1%) was never smokers, and 96.5% had a histological diagnosis of adenocarcinoma. One hundred and seven patients (53.2%) received crizotinib as the first‐line therapy at the diagnosis of metastatic NSCLC. Forty‐four patients (21.9%) had already occurred brain metastases before crizotinib treatment. The baseline characteristics of patients are described in Table 1.

**Table 1:** Demographic and baseline characteristics of all patients (n = 201)

Characteristic	n	%
Median age (range), years	50 (24‐83)	—
Gender		
Male	95	47.2
Female	106	52.8
Smoking history		
Never	139	69.1
Former/Current	62	30.9
Histological subtype		
Adenocarcinoma	194	96.5
Squamous cell carcinoma	2	1.0
Adenosquamous carcinoma	3	1.5
Large cell carcinoma	2	1.0
Stage at diagnosis		
III	16	8.0
IV	185	92.0
Line of crizotinib therapy		
1	107	53.2
≥2	94	46.8
Brain metastases prior to crizotinib		
Present	44	21.9
Absent	157	78.1
Best response to crizotinib		
CR or PR	125	62.2
SD	59	29.4
PD	17	8.4

Abbreviations: CR, complete response; PD, progressive disease; PR, partial response; SD, stable disease.

### 3.2 Efficacy of crizotinib

**Figure 1:**
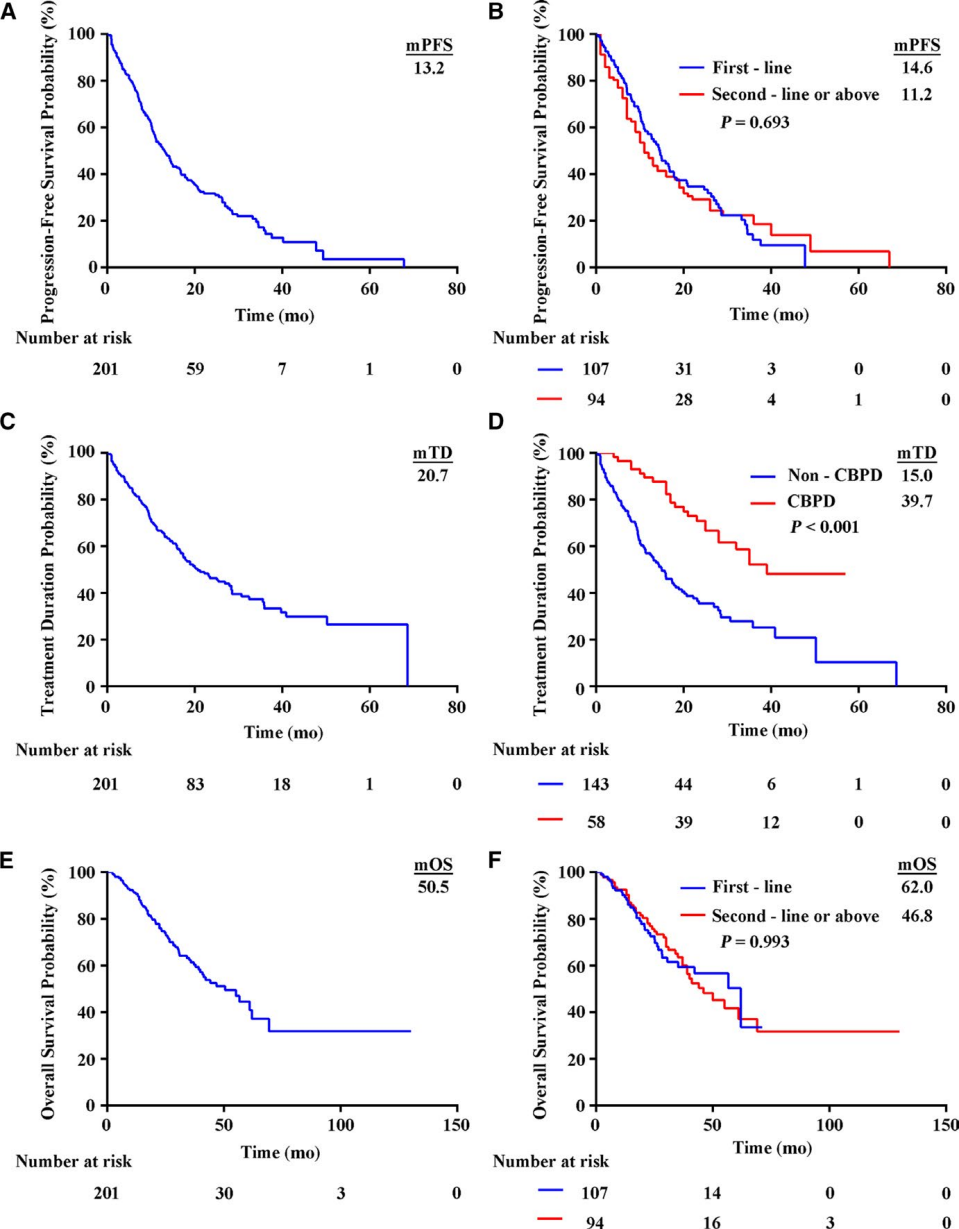
Kaplan‐Meier curves for all patients and subgroups. The median PFS1 with initial crizotinib therapy in the total cohort of patients was 13.2 months (95% CI 10.8‐15.6 months) (A). PFS1 on crizotinib therapy was not significantly different between the first‐line and second‐ or above‐line therapy groups (B). The median TD in the total patient cohort was 20.7 months (95% CI 15.1‐26.3 months) (C). TD on crizotinib in the CBPD group was significantly longer than that in the non‐CBPD group (D). The median OS in the total patient cohort was 50.5 months (95% CI 37.0‐64.0 months) (E). OS was not significantly different between the first‐line and second‐ or above‐line therapy groups (F)

In the total cohort, 125 patients (62.2%) achieved PR and 59 (29.4%) maintained SD, while 17 (8.4%) developed PD. The ORR achieved with crizotinib was 62.2% and the disease control rate was 91.5%. The median PFS1 with crizotinib treatment was 13.2 months (95% CI, 10.8 to 15.6 months) and six patients discontinued crizotinib treatment owing to adverse events (Figure 1A). One hundred and seven patients (53.2%) who received crizotinib as the first‐line therapy exhibited a median PFS1 of 14.6 months (95% CI, 11.5 to 17.7 months) while the median PFS1 of those who received the second‐ or above‐line therapy (n = 94) was 11.2 months (95% CI, 8.4 to 14.0 months). The PFS1 on initial crizotinib therapy was not significantly different between the first‐line and second‐ or above‐line groups (*P* = .693) (Figure 1B).

The median OS in the total cohort was 50.5 months (95% CI, 37.0 to 64.0 months) (Figure 1E). One hundred and seven (53.2%) patients who received crizotinib as the first‐line therapy had a median OS of 62.0 months (95% CI, 37.2 to 86.8 months) as compared with 46.8 months (95% CI, 35.1 to 58.5 months) in 94 patients who received crizotinib as the second‐ or above‐line therapy (*P* = .993) (Figure 1F). Although the median OS in the CBPD group was 61.0 months as compared with 41.4 months in the non‐CBPD group, the difference was not significantly different (*P* = .086).

### 3.3 Characteristics of CBPD patients

**Figure 2:**
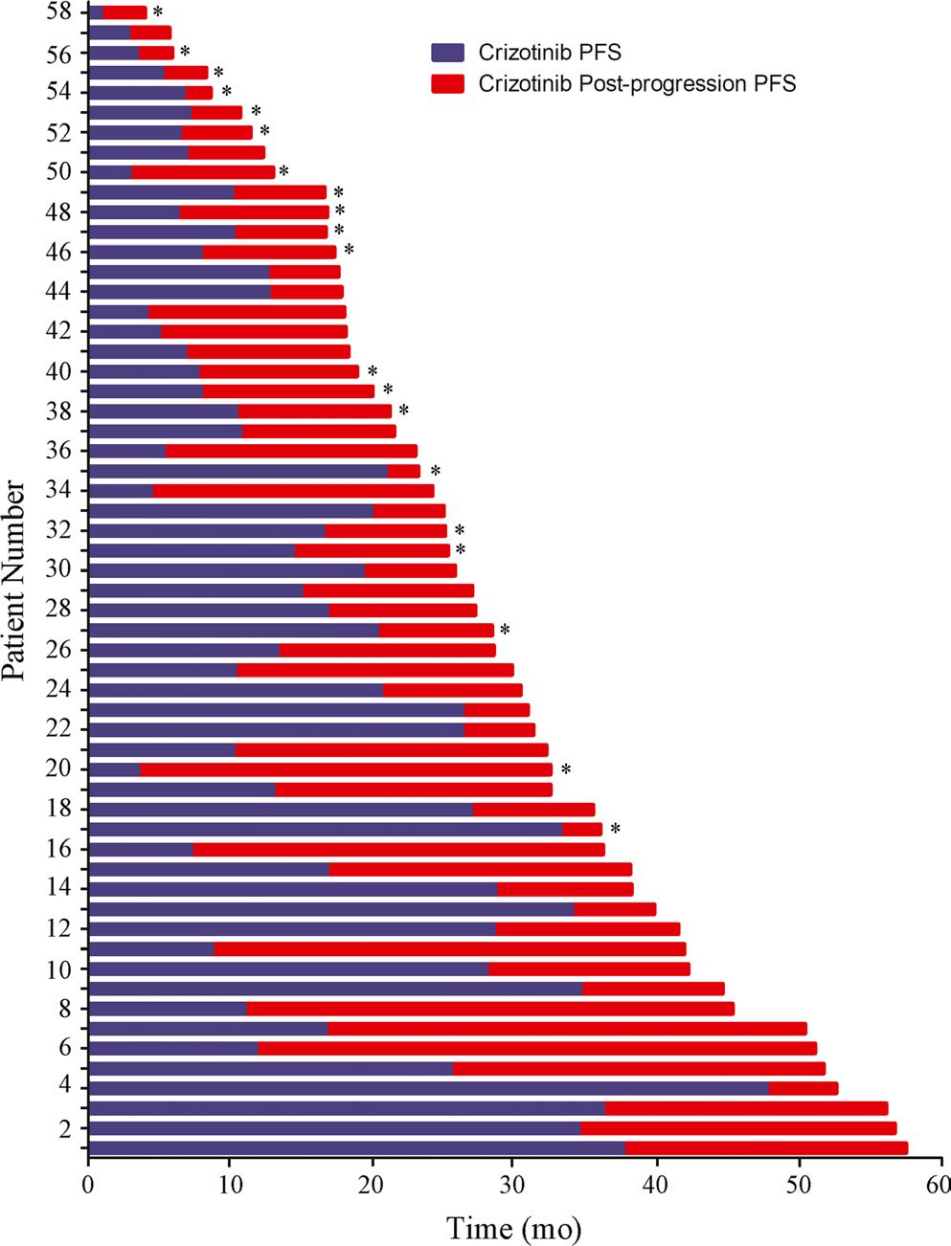
Individual swimmer plots for each patient in the CBPD group, showing PFS1 on initial crizotinib therapy (blue) and sequential post‐progression therapy (red). Patients who discontinued crizotinib and ultimately died are depicted with a “*”

A subset of 150 patients pretreated with crizotinib eventually developed PD, and 58 of these patients (38.7%) continued crizotinib therapy beyond the RECIST‐defined PD. The most common metastasis site was the brain (60.3%). In the CBPD group, 28/35 (80.0%) patients developed brain metastases and received local therapy at the time of initial RECIST‐defined PD. A total of 23 patients received palliative brain radiation, while four underwent brain tumor surgical resection. Only one patient received both brain radiation and brain tumor surgical resection. Pearson's Chi‐squared test was used to compare the significance of baseline differences between the CBPD subgroup and the non‐CBPD subgroup (Table 2). Post‐progression characteristics and the clinical outcomes of the CBPD patients are described in Table 3. The median post‐progression PFS (PFS2, defined as time between the first RECIST‐defined PD and termination of crizotinib therapy) was 10.5 months (range, 1.9‐39.3 months) in the CBPD patient group. Swimmer plots for the CBPD patients showed that continuing crizotinib therapy could still achieve ongoing clinical survival benefit (Figure 2).

**Table 2:** Baseline data differences between the CBPD and the non‐CBPD subgroup on crizotinib by Pearson's Chi‐squared test

Variables	CBPD (n = 58)	Non‐CBPD (n = 92)	*P* value
Age (years)			.561
＞60	15	20	
≤60	43	72	
Gender			.626
Male	26	45	
Female	32	47	
Smoking history			.311
Never	43	61	
Former/current	15	31	
Histological types			—
Adenocarcinoma	58	92	
Other	0	0	
Stage at diagnosis			.074
III	4	1	
IV	54	91	
Crizotinib treatment			.067
First‐line	37	40	
≥Second‐line	21	52	
Baseline CNS metastases			.758
Present	12	21	
Absent	46	71	

Abbreviation: CNS, central nervous system.

**Table 3:** Post‐progression characteristics and clinical outcomes of patients in the CBPD Group (n = 58)

Characteristic	n	%
Disease progression site		
Brain	35	60.3
Lung	11	19.0
Bone	3	5.2
Liver	5	8.6
Pleura	1	1.7
Lymph nodes	1	1.7
Multiple metastases	2	3.5
Local therapy beyond brain metastases		
Brain radiation	23	65.7
Brain tumor surgical resection	4	11.4
Combination	1	2.9
None	7	20.0
PFS2 on Crizotinib		
0 to <3 months	5	8.6
3 to <6 months	10	17.2
6 to <9 months	6	10.3
9 to <12 months	15	25.9
12 to <24 months	15	25.9
24 to <36 months	6	10.3
≥36 months	1	1.8

Abbreviations: CBPD, continuing crizotinib therapy beyond progressive disease; PFS2, time between the initial imaging evidence of PD and termination of crizotinib therapy.

### 3.4 TD analysis

**Figure 3:**
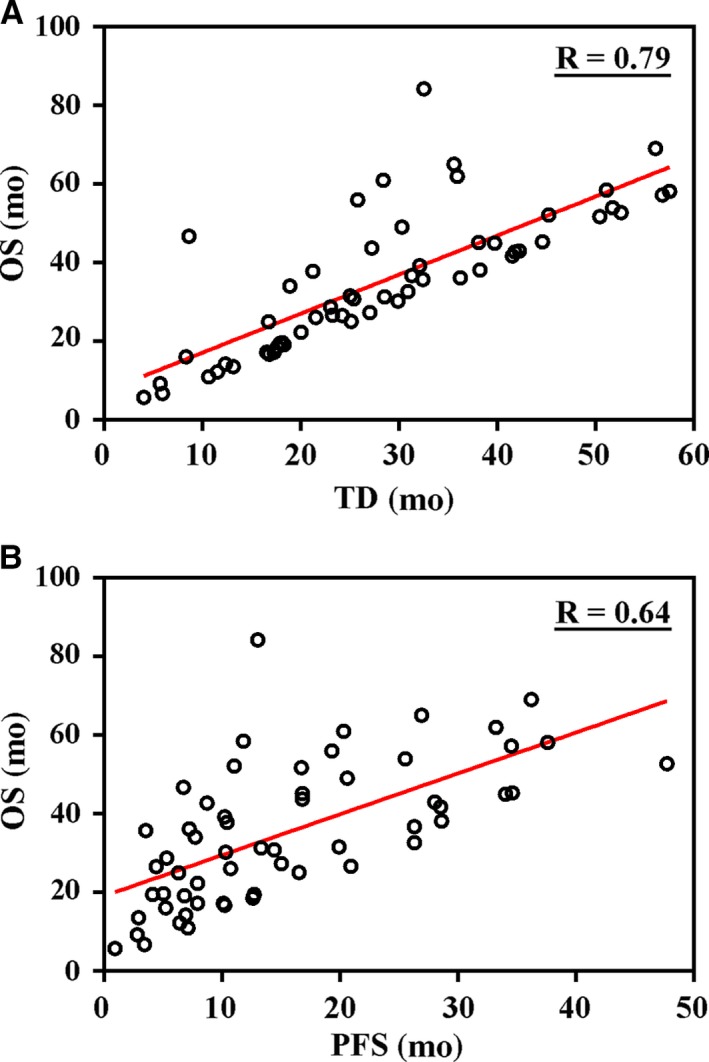
Scatter diagrams of OS in the CBPD patient group. The endpoint TD correlated better with OS (A) than with PFS (B)

Lastly, we characterized the treatment duration in this population. The median TD of crizotinib in the total patient cohort was 20.7 months (Figure 1C). TD of crizotinib in the CBPD group was significantly longer than that in the non‐CBPD group (median 39.7 vs 15.0 months, respectively;* P* < .001) (Figure 1D). To better investigate the correlation between TD and OS, we further calculate the statistical data of CBPD patients. The scatter diagrams for these patients showed that TD correlated better with OS (*R* = .79) than that with PFS (*R* = .64) (Figure 3).

## 4 DISCUSSION

In this paper, we described that TD correlated better with OS, and TD might be a more reasonable surrogate clinical endpoint than PFS to evaluate the efficacy of crizotinib in sequential therapy.

Crizotinib has been reported to achieve higher response rate and significantly longer PFS than chemotherapy in large‐scale clinical trials,^3^, ^4^ and it has been approved worldwide as a standard therapy for advanced NSCLC patients with *ALK* oncogene fusion. Invariably, like other targeted therapies, acquired resistance has emerged as the major hurdle preventing its persistent clinical efficacy. Previously, it has been reported that patients with epidermal growth factor receptor (*EGFR)* mutation‐positive NSCLC can develop a disease flare upon discontinuation of EGFR‐TKI therapy,^13^ indicating that *EGFR* mutation‐positive tumors continue to depend on EGFR signaling. Disease flare following discontinuation of crizotinib in *ALK*‐positive NSCLC has also been documented,^14^ which suggests that despite PD, TKIs could be continued in use until a new treatment is initiated. The RECIST criteria are the gold standard and a common approach for the evaluation of antitumor therapy; however, the RECIST may not be the most appropriate criteria for terminating TKI treatment.^8^, ^15^, ^16^

In the current treatment scenario, a majority of patients who are highly responsive to crizotinib treatment ultimately relapse and develop local or slowly PD typically within 1 year. Some of them have continued receiving crizotinib in combination with other locoregional therapies, and the reported outcomes have demonstrated that patients with advanced *ALK*‐positive NSCLC may derive substantial clinical benefit from continuing crizotinib beyond the RECIST‐defined PD. Ou et al^8^ reported CBPD patients had a significantly longer median OS than non‐CBPD patients from the time of PD (16.4 vs 3.9 months, *P* < .0001) and from the time of crizotinib initiation (29.6 vs 10.8 months, *P* < .0001). Multiple‐covariate Cox regression analysis revealed that CBPD remained significantly associated with improved OS after adjusting for relevant factors. In real‐world studies, the reported median PFS of crizotinib treatment has ranged from 13.3 to 17.6 months.^17^, ^18^ Liu et al^9^ reported that crizotinib continuation beyond the RECIST‐defined PD was feasible in clinical practice. The median time between the initial imaging evidence of PD and termination of crizotinib therapy (PFS2) was nearly 6.0 months. Lei et al^10^ found that patients who developed central nervous system failure and continued crizotinib treatment beyond PD could achieve a second median PFS of 6.3 months (95% CI, 2.9‐9.7 months).

Our findings are consistent with the outcomes of previously published retrospective studies.^9^, ^17^, ^18^ It showed that the median PFS1 of crizotinib was 13.2 months, and the median TD of crizotinib among CBPD patients was 39.7 months. Thus, CBPD was associated with a substantial survival benefit in patients with advanced *ALK*‐rearranged NSCLC. We observed a much longer PFS2 benefit of 10.5 months in our cohort than that previously reported value of 6.0 months. A major reason was that more than half of the CBPD patients (60.3%) in our cohort developed brain metastases during crizotinib treatment, and 80% of these intracranial PD patients received local therapies including brain radiation or brain tumor surgical resection. In addition, they kept on receiving crizotinib therapy and 74.2% of them achieved a prolonged post‐progression PFS (PFS2) exceeding 6 months, while 38% achieved a prolonged post‐progression PFS (PFS2) of more than 12 months. These outcomes further demonstrated that local therapy could do help to control disease with crizotinib in oligoprogressive disease, and TD as a clinical endpoint instead of PFS was more meaningful in evaluating the real‐world clinical efficacy of crizotinib beyond the RECIST‐defined PD.

**Figure 4:**
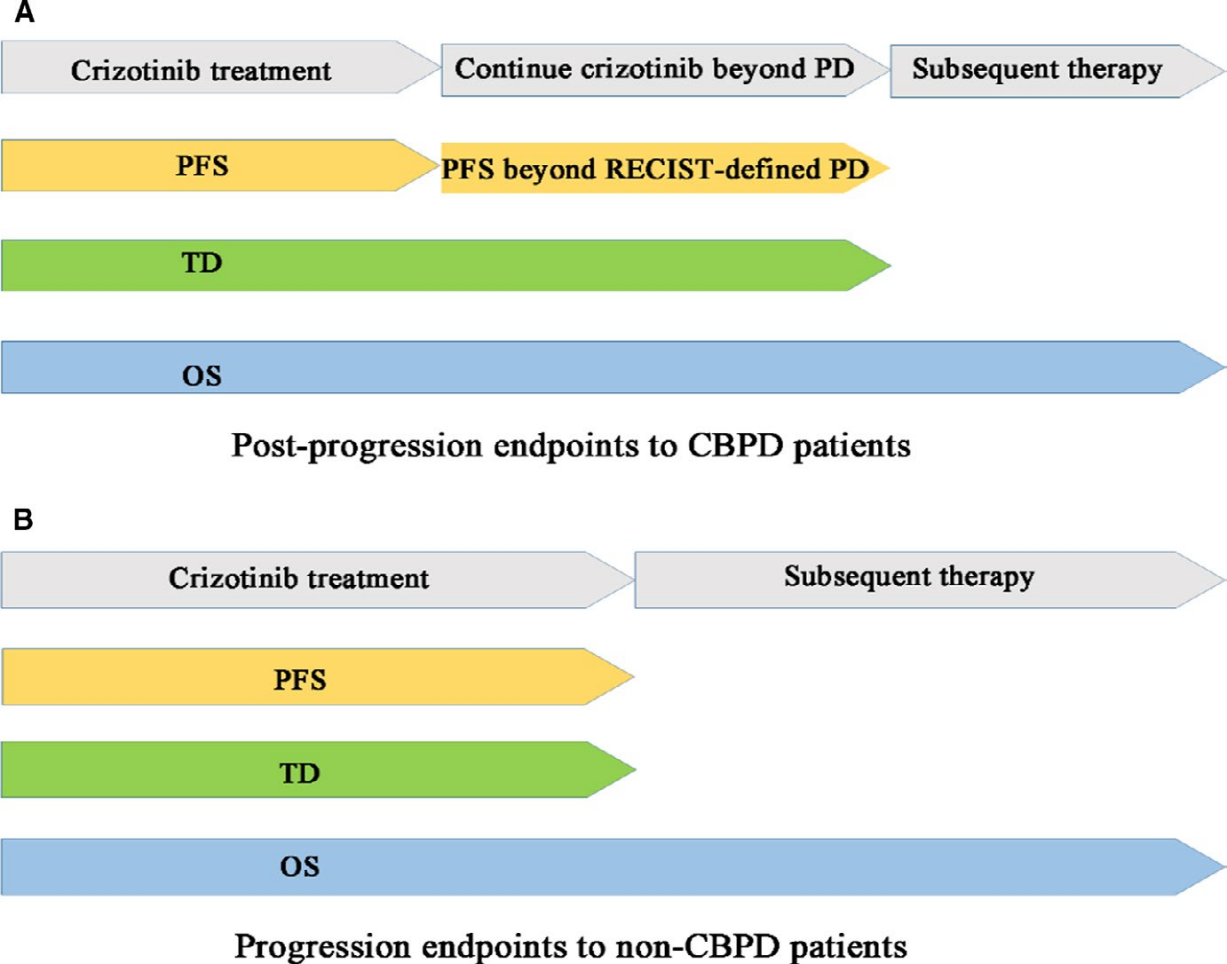
Progression and post‐progression endpoints evaluating the efficacy of crizotinib in the CBPD (A) and non‐CBPD (B) patient groups

The fact that crizotinib is often administered in multiple lines of therapy setting to patients with advanced *ALK*‐positive NSCLC is worthy of special attention. The FLAURA post‐progression study^19^ demonstrated intermediate clinical endpoints between PFS and OS further define efficacy, and can be used to assess clinically meaningful PFS improvement beyond the initial RECIST progression. The efficacy assessment mode frequently used in clinical practice—simple PFS addition with different lines of therapy—may not be able to provide an accurate efficacy evaluation and a substantially preserved PFS beyond PD. In CBPD patients, using this inflexible assessment mode is bound to artificially neglect and cut down the PFS improvement beyond PD. Nevertheless, it should also be acknowledged that not all patients pretreated with crizotinib will continue receiving it beyond PD. If we attach the PFS2 to each patient, it will unrealistically extend the efficacy of crizotinib. The progression and post‐progression endpoints evaluating the efficacy of crizotinib in CBPD and non‐CBPD patients are illustrated in Figure 4.

TD, a more pragmatic intermediate endpoint, which is defined as the time from treatment initiation to discontinuation, may be a more accurate surrogate endpoint to evaluate the efficacy of crizotinib in the post‐progression setting with multiple lines of therapy. Our study showed the median TD of crizotinib was 20.7 months, and by contrast, the median PFS1 of crizotinib was 13.2 months and the median post‐progression PFS (PFS2) was 10.5 months. TD, an endpoint which takes CBPD into consideration in the efficacy assessment, appears more reasonable and accurate to reflect the real efficacy of crizotinib among unselected *ALK‐*positive NSCLC patients. Based on the real‐world outcomes reported above, TD substituted for PFS or different PFS additions could be a more meaningful endpoint further evaluating the efficacy of crizotinib in post‐progression settings, especially among CBPD patients. Updated results from the PROFILE 1014 study indicated the median OS was not reached (95% CI, 45.8 to NR) with first‐line crizotinib therapy, as compared with 47.5 months (95% CI, 32.2 to NR) with first‐line pemetrexed‐platinum chemotherapy.^20^

There are several limitations to the present study. Firstly, it represents a single‐center retrospective analysis without verification by multicenter and large‐scale clinical trials. Secondly, the clinical information and follow‐up data of some patients in the study cohort were not well documented in their medical records. Thirdly, patient selection bias for the study is not negligible, and this may not be representative of all patients with *ALK*‐rearranged NSCLC, because the samples are scattered.

## 5 CONCLUSIONS

This retrospective, real‐world study shows good clinical efficacy of crizotinib in Chinese patients with *ALK*‐positive NSCLC. Treatment duration, as a surrogate intermediate clinical endpoint instead of PFS, may correlate better with OS and may be more accurate to evaluate the efficacy of crizotinib in sequential therapy for *ALK*‐positive NSCLC patients.

## CONFLICT OF INTEREST

The authors declare that they have no competing interests.

## AUTHOR CONTRIBUTIONS

Guangjian Yang designed and drafted the manuscript. Di Ma, Haiyan Xu, Lu Yang, Junling Li, Puyuan Xing, and Yan Wang discussed and revised the manuscript. All authors read and approved the final manuscript.

## Data Availability

The material supporting the conclusion of this study has been included in the article.
